# Sustained anti-osteoporotic action of risedronate compared to anti-RANKL antibody following discontinuation in ovariectomized mice

**DOI:** 10.1016/j.bonr.2020.100289

**Published:** 2020-06-05

**Authors:** Toshinobu Omiya, Jun Hirose, Yasunori Omata, Tsukasa Tominari, Masaki Inada, Hisato Watanabe, Takeshi Miyamoto, Sakae Tanaka

**Affiliations:** a Department of Orthopaedic Surgery, Faculty of Medicine, The University of Tokyo, Hongo 7-3-1, Bunkyo-ku, Tokyo 113-0033, Japan; b Bone and Cartilage Regenerative Medicine, The University of Tokyo, 7-3-1 Hongo, Bunkyo-ku, Tokyo 113-8655, Japan; c Department of Biotechnology and Life Science, Tokyo University of Agriculture and Technology, 2-24-16 Nakacho, Koganei-shi, Tokyo 184-8588, Japan; d Department of Orthopedic Surgery, Keio University, School of Medicine, 35 Shinano-machi, Shinjuku, Tokyo 160-8582, Japan

**Keywords:** Bisphosphonate, Anti-RANKL antibody, Discontinuation, Ovariectomized mice, Bone morphometric analysis

## Abstract

Bisphosphonates and the anti-receptor activator of nuclear factor-kappa B ligand (RANKL) antibody denosumab are effective anti-resorptive drugs commonly prescribed for osteoporosis. Both drugs may, however, have intolerable side effects; so, it is critical to examine their residual efficacy such as maintenance of bone mass following cessation. Therefore, we compared the changes in bone histology following discontinuation of the aminobisphosphonate risedronate and anti-RANKL antibody in ovariectomized (OVX) mice. Twelve-week-old female C57BL/6 N mice were OVX or sham operated. Four weeks after surgery, mice were treated with vehicle, a single injection of anti-RANKL antibody (5 mg/kg), or risedronate (5 μg/kg/day, s.c.) for 4 weeks (the treatment period), followed by vehicle treatment for an additional 4 weeks (discontinuation period). The lumbar spine and proximal tibia were evaluated by micro-computed tomography. In addition, the lumbar spine, proximal tibia, and the femoral shaft were examined by bone histomorphometry. After 4 weeks of discontinuation, OVX mice initially treated with the anti-RANKL antibody exhibited a trend of bone loss associated with increased turnover in both trabecular and cortical bones, although the difference was not significant. By contrast, OVX mice treated with risedronate exhibited maintained or even increased bone mass and suppressed bone turnover. Patients discontinuing denosumab should be carefully monitored for recurrent osteoporosis symptoms, and a replacement drug should be considered.

## Highlights

• Bone mass and suppression of turnover were maintained after stopping bisphophonates.
• Bone turnover was rapidly increased after discontinuation of anti-RANKL antibody.
• Periosteal bone formation was maintained after administration of antiresorptive drugs.

## 1 Introduction

Osteoporosis is characterized by low bone mass and elevated risk of fragility fractures. Recent advances in the understanding of bone metabolism have led to the development of various anti-osteoporosis drugs. Bisphosphonates (BPs) are the most commonly used anti-resorptive drugs because they demonstrated efficacy for increasing bone mineral density (BMD) and reducing fracture risk (Liberman et al., 1995; Black et al., 1996; Harris et al., 1999). After administration, BPs are incorporated into bone and released during resorption by osteoclasts, leading to sustained suppression of bone remodeling. Denosumab, a fully human monoclonal antibody against receptor activator of nuclear factor-kappa B ligand (RANKL), is another anti-resorptive drug with a distinct mechanism of action. Denosumab increases BMD and reduces the risk of fragility fractures by inhibiting the differentiation and activation of osteoclasts (Cummings et al., 2009).

Although both of these anti-resorptive agents are effective and recommended for osteoporosis treatment by various guidelines, their adverse effects, such as osteonecrosis of the jaw and atypical femoral fractures, have been reported (Goh et al., 2007; Lenart et al., 2008). Therefore, these drugs may be discontinued temporarily or permanently after several years of administration (Recker et al., 2009; Watts and Diab, 2010). In such cases, it is important to know how rapidly the anti-osteoporotic efficacy is lost (i.e., how quickly low BMD returns). However, this rate may vary among medications, as several studies have reported different clinical outcomes following discontinuation of BPs or denosumab. For instance, the Fracture Intervention Trial Long-term Extension (FLEX) study demonstrated that among postmenopausal women who had used the BP alendronate for 5 years, those randomized to receive a placebo for an additional 5 years had rates of non-vertebral and morphometric vertebral fractures similar to those randomized to receive an additional 5 years of alendronate (Black et al., 2006). On the other hand, a rapid decrease in BMD (McClung et al., 2017) and concomitant increase in the incidence of multiple lumbar spine fractures (Cummings et al., 2018; Anastasilakis and Makras, 2016) were reported after discontinuation of denosumab, suggesting that rapid switching to another medication such as a bisphosphonate may be required. However, there is still limited information regarding the effects of BP and anti-RANKL antibody discontinuation on BMD and other parameters of bone integrity. Moreover, the changes in bone histology following the discontinuation of these drugs remain largely unknown. There are two representative BPs: alendronate and risedronate. Risedronate is a nitrogen-containing third-generation BP, and we have previously investigated and reported the mechanism of its anti-resorptive effects (Matsumoto et al., 2011). To further investigate the characteristics of risedronate, it was selected as the BP in the present study. The purpose of this study was to evaluate the histological changes in cancellous and cortical bone resulting from discontinuation of the anti-RANKL antibody and the aminobisphosphonate risedronate in ovariectomized (OVX) mice.

## 2 Materials and methods

### 2.1 Reagents and animals

Risedronate was provided by EA Pharma Co. (Tokyo, Japan), and the anti-mouse RANKL monoclonal antibody (OYC1) was purchased from Orient Yeast Co. (Tokyo, Japan). Twelve-week-old virgin female C57BL/6 N mice were purchased from Sankyo Labo Service Co. (Tokyo, Japan). All mice were housed under specific pathogen-free conditions, controlled temperature, controlled humidity, and a 12-h/12-h light/dark cycle, with ad libitum access to food and water. All animal handling and experimental protocols were conducted in accordance with the directives of the Animal Care and Use Committee of The University of Tokyo.

### 2.2 Treatment protocols

**Fig. 1:**
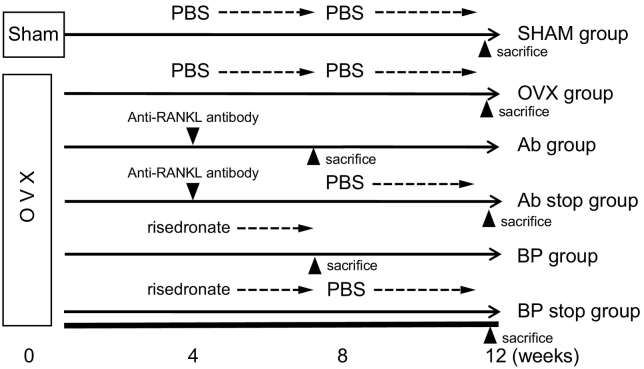
Experimental protocol. Mice were first randomly separated to receive ovariectomy or sham surgery (SHAM group). The ovariectomized mice were then randomly divided into 5 treatment groups (*n* = 4–6) as described in the methods: OVX, Ab, Ab stop, BP, and BP stop. Mice in the Ab and BP groups were sacrificed at 8 weeks after surgery, and the other mice were sacrificed at 12 weeks after surgery.

A schematic of the study design is shown in Fig. 1. Twenty-eight mice were randomly assigned to receive OVX (*n* = 22) or sham surgery (SHAM, *n* = 6). Four weeks after surgery, the OVX mice were further subdivided into the following treatment groups: subcutaneous (s.c.) phosphate-buffered saline (PBS) injection for 8 weeks (OVX group, n = 6), the anti-RANKL antibody (single s.c. injection of 5 mg/kg) (Ab group, *n* = 4), the anti-RANKL antibody (single s.c. injection of 5 mg/kg) followed 4 weeks later by PBS injection (s.c.) for 4 weeks (Ab stop group, n = 4), risedronate (5 μg/kg/day, s.c.) for 4 weeks (BP group, n = 4), and risedronate (5 μg/kg/day, s.c.) for 4 weeks followed by s.c. PBS for 4 weeks (BP stop group, n = 4). The anti-RANKL antibody dose regimen is equivalent to once every 6 months in humans. Mice in the SHAM, OVX, Ab stop, and BP stop groups were euthanized 12 weeks after surgery. Mice in the Ab and BP groups were euthanized 8 weeks after surgery. Hind limbs and lumbar spines were subjected to micro-CT and histomorphometric analyses.

### 2.3 Micro-CT analysis

The proximal tibia and lumbar spine were imaged using a Scan Xmate-L090 Scanner (Comscantechno Co., Ltd., Yokohama, Japan). Three-dimensional microstructural imaging data were reconstructed and structural indices calculated using TRI/3D-BON software (RATOC System, Osaka, Japan). Bone volume (BV), tissue volume (TV), trabecular number (Tb.N), trabecular thickness (Tb.Th), and trabecular separation (Tb.Sp) of trabecular bone were measured. BV/TV was calculated.

### 2.4 Histomorphometric analysis

Histomorphometric analysis was performed at 400× magnification on undecalcified sections from the secondary spongiosa area of the proximal tibia and the lumbar spine. Cortical bone measurements were performed in the femoral diaphysis and lumbar spine. For analysis of bone mineralization, mice were injected (s.c.) with 20 mg/kg tetracycline hydrochloride 5 days and with 16 mg/kg calcein 2 days before they were sacrificed. The eroded surface (ES), osteoclast surface (Oc.S), and osteoblast surface (Ob.S) were measured. The eroded surface/bone surface ratio (ES/BS), osteoclast surface/bone surface ratio (Oc.S/BS), osteoblast surface/bone surface ratio (Ob.S/BS), and mineral apposition rate (MAR) were calculated. The balance of bone metabolism in the proximal tibia as the %ratios of osteoid surface/bone surface (OS/BS), ES/BS, and quiescent surface/bone surface (QS/BS) were calculated.

### 2.5 Statistical analysis

The results are expressed as the mean ± standard deviation. Group means were compared by analysis of variance with post hoc Tukey's honest significant difference tests for pair-wise comparisons. A *p* < .05 (two-tailed) was considered to be statistically significant for all tests. All analyses were conducted using JMP®12 (SAS Institute Inc., Cary, NC, USA) and GraphPad Prism 8.4.1 (Graphpad Software, San Diego, USA).

## 3 Results

### 3.1 Sustained anti-osteoporotic action of risedronate compared with anti-RANKL antibody

**Fig. 2:**
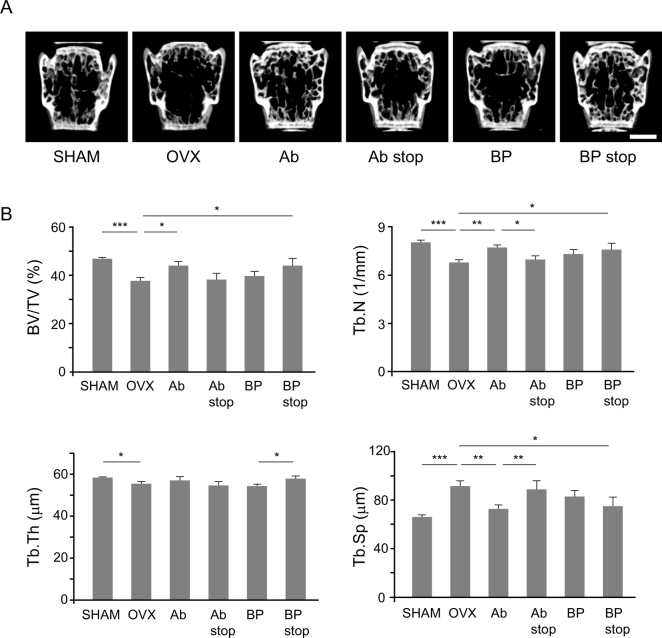
Micro-CT analysis of the lumbar spine at 4 weeks after randomization to Ab and BP groups and after 8 weeks in Sham, OVX, Ab stop, and BP stop groups. (A) Representative micro-CT images of the lumbar spine in each group. Scale bar: 1 mm. (B) Micro-CT analysis parameters of the lumbar spine in each group of trabecular bone. Parameters: bone volume/tissue volume (BV/TV, %), trabecular number (Tb.N, 1/mm), trabecular thickness (Tb.Th, μm), and trabecular separation (Tb.Sp, μm). *, *p* < .05; **, *p* < .01; ***, *p* < .001.

**Fig. 3:**
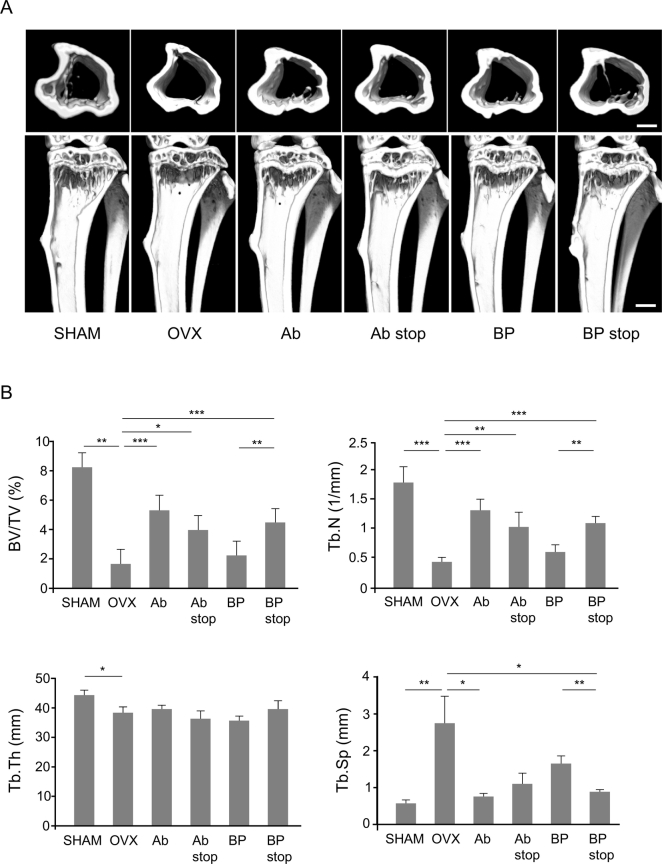
Micro-CT analysis of the proximal tibia at 4 weeks after randomization to Ab and BP groups and after 8 weeks in Sham, OVX, Ab stop, and BP stop groups. (A) Representative micro-CT images of the proximal tibia in each group. Scale bar: 500 μm. (B) Micro-CT analysis parameters of the proximal tibia in each group. Parameters are defined in Fig. 2. *, *p* < .05; **, *p* < .01; ***, *p* < .001.

In the OVX group, BV/TV, Tb.N, and Tb.Th were significantly lower and Tb.Sp was higher than those in the SHAM group (Fig. 2B). This demonstrated that OVX had successfully been performed. We compared the effects of anti-RANKL antibody (Ab) administration and cessation to risedronate (bisphosphonate, BP) administration and cessation on bone structure by micro-CT analysis of the lumbar spine (Fig. 2A) and proximal tibia (Fig. 3A). In the lumbar spine, the Ab group showed significantly higher BV/TV and Tb.N and lower Tb.Sp compared with the OVX group 4 weeks after single Ab administration. Alternatively, these three parameters did not differ significantly between the OVX and Ab stop groups 8 weeks after Ab administration. BV/TV was not significantly different between the Ab group and the Ab stop group (*p* = .057). Tb.N was significantly lower while Tb.Sp was significantly higher in the Ab stop group than in the Ab group. By contrast, there were no significant differences in these parameters between the OVX group and either the BP group or the BP stop group. Unexpectedly, Tb.Th was significantly higher in the BP stop group than in the BP group. In the BP stop group, the BV/TV, Tb.N, and Tb.Th were higher and Tb.Sp was lower than those in the Ab stop group; however, the difference was not significant (Fig. 2B).

Fig. 3A shows representative micro-CT images of the proximal tibia in each group. In the Ab group, BV/TV and Tb.N were significantly higher while Tb.Sp was significantly lower than in the OVX group 8 weeks after surgery, similar to results in the lumbar spine. Both BV/TV and Tb.N decreased after Ab discontinuation (Ab stop group); however, the differences were not significant compared with those in the Ab group, and BV/TV and Tb.N remained significantly higher than in the OVX group. Alternatively, BV/TV and Tb.N were significantly higher in the BP stop group than in the BP group. There was no significant difference in all these parameters between the Ab stop and BP stop groups (Fig. 3B).

### 3.2 Histomorphometric parameters of the proximal tibia

**Fig. 4:**
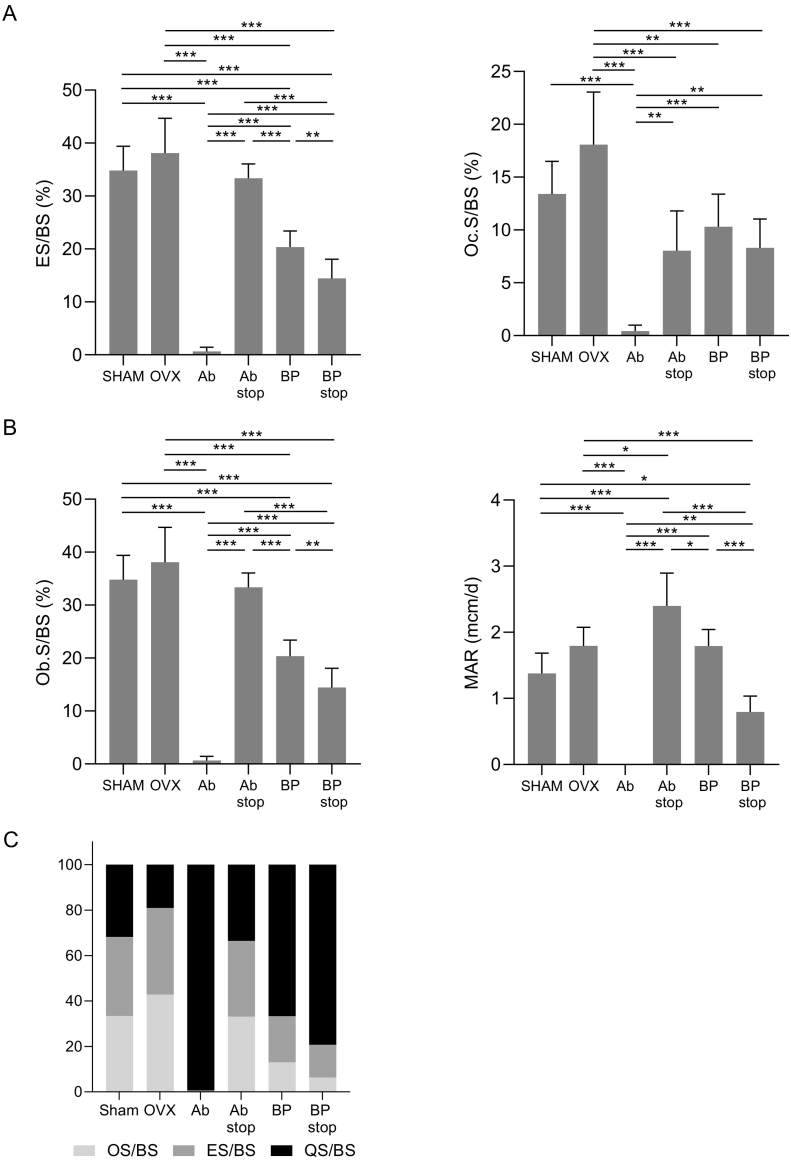
Bone histomorphometry of the proximal tibia secondary spongiosa area at 8 weeks after randomization. (A) Bone resorption parameters of the proximal tibia. Histomorphometric parameters: eroded surface/bone surface (ES/BS, %) and osteoclast surface/bone surface (Oc.S/BS, %). **, *p* ≤ .01, ***, *p* ≤ .001. (n = 4–6) (B) Histomorphometric parameters: osteoblast surface/bone surface (Ob.S/BS, %) and mineral apposition rate (MAR, μm/day). *, *p* ≤ .05, **, *p* ≤ .01, ***, *p* ≤ .001. (C) Ratio (%) of osteoid surface per bone surface (OS/BS), eroded surface per bone surface (ES/BS), and quiescent surface per bone surface (QS/BS) of proximal tibia in each group (n = 4–6).

The bone resorption parameter ES/BS was significantly higher in the Ab stop than in the Ab (*p* ≤ .001), and was also significantly lower in the BP stop group than in the BP (*p* ≤ .01) (Fig. 4A). The similar tendency was observed in other bone histometry parameters such as Oc.S/BS, ObS/BS and MAR (Fig. 4A and B).

In the comparison between Ab stop and BP stop, the ES/BS was lower in the BP stop group than in the Ab stop group (Fig. 4A). The Oc.S/BS was significantly suppressed in both Ab stop and BP stop groups compared to OVX group which did not receive any treatment; however, there was no difference between the Ab stop and BP stop groups (Fig. 4A). Bone formation parameters, such as Ob.S/BS and MAR were markedly suppressed in the BP stop group, reflecting continued suppression of bone turnover by risedronate. These parameters in the BP stop group were significantly lower those than in the Ab stop group (Fig. 4B). Fig. 4C shows the balance of bone metabolism in proximal tibia as the % ratios of OS/BS, ES/BS, and QS/BS. Both OS/BS and ES/BS were lower in the BP stop group than in all other groups, demonstrating that the suppression of bone turnover was maintained even after discontinuation of risedronate (Fig. 4C).

### 3.3 Histomorphometric analysis of cortical bone in the femoral shaft and the lumbar spine

**Fig. 5:**
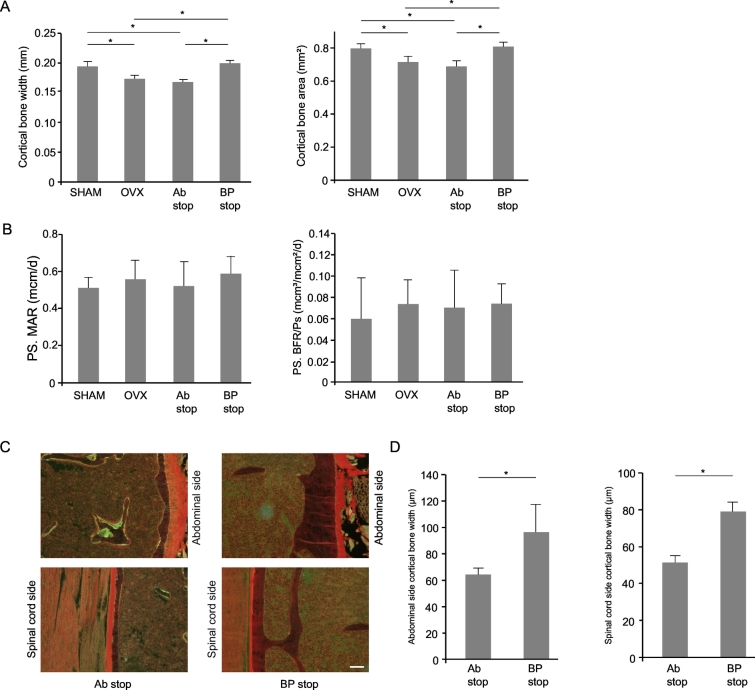
Bone histomorphometry of the femoral shaft at 8 weeks after randomization. (A) Histomorphometric parameters: cortical bone width (mm) and cortical bone area (mm^2^). *, *p* < .05. (n = 4–6). (B) Histomorphometric parameters: mineral apposition rate in periosteum (Ps. MAR, μm/day) and bone formation rate in periosteum/periosteal surface (Ps. BFR/Ps, mm^3^/mm^2^/year). (n = 4–6). (C) Representative images of lumbar spine cortical bone with Villanueva Goldner stain. Scale bar: 20 μm. (D) Cortical bone width (μm) of the abdominal and spinal cord sides of the lumbar spine. *, *p* < .05. (n = 4).

We then performed a histomorphometric analysis of cortical bone. Both cortical bone width and area were significantly lower in the Ab stop group than in the SHAM group (Fig. 5A). By contrast, these markers were significantly higher in the BP stop group than in the OVX and Ab stop groups. There were no significant differences in bone formation parameters on the periosteal surface among groups (Fig. 5B). In the lumbar spine, cortical bone width was significantly higher in the BP stop group than in the Ab stop group at both the abdominal and spinal cord sides of the spine (Fig. 5C and D).

## 4 Discussion

Clinical studies have shown that the skeletal response differs between bisphosphonate and denosumab discontinuation, but little is known about the changes in bone metabolism during the post-treatment period. In the present study, we investigated these effects of discontinuation in OVX mice by micro-CT and histomorphometric analyses. Micro-CT analyses of the lumbar spine and proximal tibia showed that the increased bone mass was observed 4 weeks after single anti-RANKL antibody treatment. There was a trend toward diminished BV/TV after an additional 4-week treatment-free period, although the difference was not statistically significant. By contrast, the increase in BV/TV did not reach statistical significance after 4 weeks of BP treatment compared with that in the OVX group but was higher, especially in proximal tibia, 4 weeks after discontinuation. These results are consistent with clinical observations that the anti-osteoporotic efficacy of BP gradually increases during treatment and is sustained even after cessation because the drug is incorporated into bone and gradually released during resorption (Diab and Watts, 2013; McClung et al., 2013). Several clinical studies have reported sustained clinical efficacy for at least several years after BP discontinuation (Black et al., 2006; Black et al., 2015; Naylor et al., 2018). By contrast, rapid recovery and overshooting of bone turnover and substantial bone loss have been observed after discontinuation of denosumab (Popp et al., 2018; Roux et al., 2019; Zanchetta et al., 2018). Our data did not show the significant bone loss after discontinuation of anti-RANKL antibody, although there were trends toward decreased bone mass. In contrast, BV/TV was maintained or even increased after discontinuation of BP. These data provide histological evidence for this difference in bone quantity change after discontinuation of BP and anti-RANKL antibody treatment.

We then investigated the change in bone turnover after stopping risedronate and anti-RANKL antibody treatment by histomorphometric analysis of trabecular bone in the proximal tibia. The ES/BS, which indicates the bone resorption activity of osteoclasts, was significantly lower in mice withdrawn from BP (BP stop group) than BP groups and was also significantly lower than Ab stop group, whereas the ES/BS in the Ab stop group was significantly higher than in the Ab group. Besides, it was comparable between mice withdrawn from the anti-RANKL antibody (Ab stop group) and the untreated OVX group. Similarly, bone formation as evaluated by MAR was still significantly suppressed in the BP stop group compared with that in the BP group and also in the Ab stop, on the other hand, it was enhanced in the Ab stop group compared with Ab group. In parallel with the rapid loss of bone mass after cessation of the anti-RANKL antibody, a rebound in bone turnover markers (‘overshooting’) has also been reported (Bone et al., 2011; Brown et al., 2013; Miller et al., 2008). Consistent with these studies, both bone resorption by osteoclasts and bone formation by osteoblasts returned to control levels within weeks of anti-RANKL antibody discontinuation. Although some recovery of bone turnover markers after BP discontinuation has also been reported, its extent varied depending on a specific BP agent and duration of administration. Grey et al. reported 5 years of anti-resorptive activity after a single dose of zoledronate (Grey et al., 2012). By contrast, Eastell et al. (Eastell et al., 2011) and Watts et al. (Watts et al., 2008) reported increased bone turnover marker levels one year after cessation of risedronate treatment. Fuchs et al. reported that alendronate induced more persistent suppression of bone turnover than risedronate did after treatment withdrawal (Fuchs et al., 2008). In the current study, we found that bone turnover was still suppressed 4 weeks after risedronate treatment had been stopped. Overall, these results indicate that the inhibitory effect of BPs on bone turnover is maintained longer than that of the anti-RANKL antibody in OVX mice.

We previously reported differential effects of anti-osteoporosis drugs on trabecular and cortical bone (Omiya et al., 2018; Tokuyama et al., 2015). Therefore, we also performed a histomorphometric analysis on the periosteal surfaces of the femoral diaphysis and cortical bone of lumbar vertebrae. Cortical bones of both the femoral shaft and the lumbar spine were significantly thicker after risedronate discontinuation compared with after anti-RANKL antibody discontinuation, indicating that this BP also has long-lasting effects on cortical bone after treatment stoppage. It should be emphasized that there were no differences in osteoblastic parameters of the periosteum among groups. Bone formation can occur either as uncoupled bone modeling or as part of bone remodeling. Although bone modeling is usually limited after adolescent, there are evidences that suggest that it continues, presumably to a lesser extent, in adulthood (Epker and Frost, 1966; Hattner et al., 1965). Several reports suggested that denosumab suppresses remodeling-coupled bone formation but not modeling-based bone formation on the periosteal side of cortical bone (Omiya et al., 2018; Ominsky et al., 2015). This preservation of modeling-based bone formation may partly contribute to the sustained increase in BMD by continuous denosumab treatment. Our results suggest that bone formation on the periosteal side of cortical bone is less affected than that of trabecular bone by OVX or administration of anti-resorptive drugs.

Although the present study examined an animal model of postmenopausal bone changes and cannot be directly applied to humans, the results are in qualitative agreement with clinical trials comparing BP and denosumab discontinuation. Therefore, these findings may explain the mechanisms underlying the differential effects of BP and anti-RANKL antibody discontinuation on trabecular and cortical bones. Our data histologically demonstrated the rapid increase of bone turnover after anti-RANKL antibody discontinuation, which was not observed after BP discontinuation. This further indicates the need for alternative treatment, such as BPs, following denosumab discontinuation. Future studies are required to clarify the mechanisms that occur following the discontinuation of BPs and anti-RANKL antibody in human bones.

## 5 Conclusion

OVX mice initially treated with the anti-RANKL antibody exhibited increased turnover in both trabecular and cortical bones, whereas those treated with risedronate exhibited maintained or even increased bone mass and suppressed bone turnover after discontinuation of treatment. Patients discontinuing denosumab should be carefully monitored for recurrent osteoporosis symptoms, and a replacement drug should be considered.

## Transparency document

Transparency document. Image 1

## Credit author statement

TO, TT, MI, and HW, performed the experiments. JH and YO interpreted the data. TM and ST conceived, directed, and supervised the study. All authors reviewed the manuscript.
